# Surgical Clinic on Injuries of the Elbow-Joint

**Published:** 1863

**Authors:** E. Andrews


### SURGICAL CLINIC ON INJURIES OF THE ELBOW-JOINT.

By Professor E. ANDREWS.

GENTLEMEN:—I present before you this morning two severe cases of compound and comminuted fracture and one case of simple fracture of the bones constituting the elbow-joint.

We will take the simple fracture first, as being the easiest understood, and then continue our investigations upon the severer injuries:—

You perceive that this little girl has some injury to the arm, whose nature is not obvious at first glance. She fell to-day upon her right elbow, which is now somewhat swollen and tender, yet there is no very obvious deformity on inspection, and the pain is not severe; but as diagnosis of injuries of the elbow are of great importance and sometimes rather difficult, I have placed her under the influence of ether to enable me, without pain, to make a thorough examination. Gentlemen, if in examining injuries of the elbow you fix your attention upon four anatomical landmarks, you will be guided by them to sure and infallible conclusions, and this complex subject will become to your mind simple, easy, and beautiful; but if you disregard them, and fumble indefinitely around the swollen member, trying to judge by its general form and position, you will always be in doubt and never can be sure of a good diagnosis.

The four landmarks of the elbow-joint are

1st. The olecranon process,
2d. The internal condyle,
3d. The external condyle,
4th. The button-head of the radius.

These points can always be distinguished, even in swollen limbs, except the head of the radius, and that, if it cannot be discovered, is proved to be in its own proper place, the only spot about the elbow where so large an object as the button-head can lie hid. The principle which will guide your judgment in the use of these landmarks may be clearly stated in two sentences By comparing these four bony points in the injured and in the sound limb you will ascertain

1st. If they have all the correct relative distances and directions from each other; then, there is no dislocation.

2d. If each one is firmly attached to the shaft of its own bone; then, there is no fracture.

If, on the contrary, the relative positions are altered or the landmarks move on their shafts, there is a dislocation or fracture, or both. By considering the subject in this simple and clear manner, your diagnosis will be stamped with certainty, and all other symptoms be fully accounted for.

In the present instance, I perceive that the olecranon process is somewhat drawn upward and separated by a gap from the ulna, proving a fracture. The other parts of the joint are all in normal relative position. For treatment in this case, I recommend a straight anterior splint to keep the forearm extended, so as to bring up the ulna against the fragment of the olecranon, and a figure-of-eight bandage to gently draw the process downward against the ulna.

The other two cases before us are more serious in their character, and endanger the lives of the patients. Observe now, the two crushed elbows: the flesh is ragged and torn, the bones are comminuted, and the fragments protruding through the wounds. These illustrate the class of injuries produced by railroad accidents. The patients are somewhat cold with the nervous shock, and show strong marks of suffering on their countenances, and the pulse is rather small and feeble. The injuries, therefore, take serious hold on the powers of life. At the same time, you observe they are both muscular and vigorous looking men. There is no mark of intemperance in the countenance, and the complexion is clear, strong, and healthy, showing a firm, vigorous nutrition of the skin. All these things lead us to hope that the patients have a good plastic diathesis, and will bear up well against the coming effects of their injuries. If, on the contrary, we found the skin unusually thin and delicate, or puffy and bloated with intemperance, or dotted with weak aplastic-looking festers, it would show an aplastic diathesis, and give reasons to fear that the patients would succumb too readily to their injuries. All these minor points you will observe in your patients at first inspection, in order to estimate their probable resistence to death. The next question is: can the limbs be saved? Here, you are not to judge by the amount of superficial laceration, nor yet by the complications of the fracture, but by observing whether the vital functions of the limb are still carried on.

In these cases I observe that both patients preserve a good pulse at the wrist, and, to some extent, the sensitiveness of the fingers. One of them has lost the feeling in the little finger and adjoining side of the ring finger, indicating the destruction of the ulnar nerve. The rule adopted by all sound surgeons in this accident, is to preserve the limb, if there is circulation of the blood sufficient within it to give a tolerable hope of avoiding gangrene. It is not considered justifiable to amputate for a crushed elbow, while there is a pulse at the wrist, or any other clear signs of continued circulation in the hand, for the following reasons:—

1st. The elbow-joint is not so large that a suppurative inflammation of its surfaces will very greatly endanger life. The effects will, it is true, be severe, but they are not generally fatal, as they are in a crushed knee, and the risk, therefore, may rationally be encountered.

2d. Even if mortification should occur, it does not, generally, terminate in death, as it does in the inferior extremity, and secondary operations upon the arm, although not as successful as primary, are still very well borne.

3d. The value of an arm is much greater than that of a leg. Artificial limbs serve a very good purpose for walking, but for loss of an arm no efficient mechanical substitute can be offered. It is the duty and the right, therefore, of a patient to run some risk of his life for the sake of a member which carries within itself so much of his personal usefulness and comfort. I decide, therefore, against any primary amputation.

I shall now give an anesthetic, and proceed to remove carefully all loose fragments of bone which appear to have lost their vascular connections, and shall then place the limbs upon angular splints. By to-morrow, the shock of the nervous system will have passed away, and the reaction, which precedes inflammation, will have fully set in. I shall then use cold water dressings, graduating the amount of cold according to the severity of the vascular action. The joints, in these cases, always inflame severely, and a great amount of swelling usually occurs. Following this, we shall have copious suppuration, with gangrene of such portions of flesh as are much crushed, and, probably, necrosis of some points and pieces of bone, all of which complications must be treated on the usual principles, as they arise.

Ultimately, the position of the forearm will become an important point of consideration. The joint, in such cases, must be considered, generally, destined to anchylosis, and, therefore, the member must be placed in such a degree of flexion as will render the loss of motion least injurious. This position is found, by experience, to be the one-half way between complete extension and a right angled flexion. I shall, therefore, set the splint to an angle of 135 degrees. I said, that the limb must be looked upon as *probably*, but not certainly, destined to anchylosis. Such is the truth in the case. It is my duty, therefore, on the one hand, to give the splint such an angle as to render the limb as useful as possible after the stiffening; but, on the other hand, every effort must be put forth to prevent a complete loss of mobility. With this view, I shall commence, as soon as the danger of gangrene is past, to make daily motions with the limb, so as to elongate and loosen the new-formed bands of plastic lymph, which will connect the articular surfaces. In this way, we may be able to preserve a portion of the motions of the joint, and very much increase the usefulness of the hand and forearm.

